# Taurine depletion during fetal and postnatal development blunts firing responses of neocortical layer II/III pyramidal neurons

**DOI:** 10.3389/fnmol.2022.806798

**Published:** 2022-11-17

**Authors:** Yasushi Hosoi, Tenpei Akita, Miho Watanabe, Takashi Ito, Hiroaki Miyajima, Atsuo Fukuda

**Affiliations:** ^1^Department of Neurophysiology, Hamamatsu University School of Medicine, Hamamatsu, Japan; ^2^First Department of Medicine, Hamamatsu University School of Medicine, Hamamatsu, Japan; ^3^Division of Health Science, Department of Basic Nursing, Hamamatsu University School of Medicine, Hamamatsu, Japan; ^4^Department of Biosciences and Biotechnology, Fukui Prefectural University, Fukui, Japan

**Keywords:** taurine, neocortex, pyramidal neurons, action potentials, taurine transporter-knockout mouse

## Abstract

Fetal and infant brains are rich in maternally derived taurine. We previously demonstrated that taurine action regulates the cation-chloride cotransporter activity and the differentiation and radial migration of pyramidal neuron progenitors in the developing neocortex of rodent fetuses. Here we examined the effects of fetal and infantile taurine depletion caused by knockout of the taurine transporter Slc6a6 on firing properties of layer II/III pyramidal neurons in the mouse somatosensory cortex at 3 weeks of postnatal age, using the whole-cell patch-clamp technique. The membrane excitability under resting conditions was similar between the neurons in knockout mice and those in wildtype littermates. However, the frequency of repetitive spike firing during moderate current injection was significantly lower, along with lower membrane voltage levels during interspike intervals in knockout neurons. When strong currents were injected, by which repetitive firing was rapidly abolished due to inactivation of voltage-gated Na^+^ channels in wildtype neurons, the firing in knockout neurons lasted for a much longer period than in wildtype neurons. This was due to much lower membrane voltage levels during interspike intervals in knockout neurons, promoting greater recovery of voltage-gated Na^+^ channels from inactivation. Thus, taurine depletion in pyramidal neurons blunted neuronal responses to external stimuli through increasing the stability of repetitive firing, presumably mediated by larger increases in membrane K^+^ conductance during interspike intervals.

## Introduction

The central nervous system during fetal and postnatal development is rich in maternally supplied taurine. Taurine is supplied to fetuses from maternal blood through the placenta and to offspring after birth through breast milk (Miyamoto et al., 1988; Sturman, 1993). The taurine transporter SLC6A6 (TauT) is responsible for its transport into the body and cells. As the action of taurine in the central nervous system, activation of GABA_A_, GABA_B_, and glycine receptors as a partial agonist has been known (Kilb and Fukuda, 2017). Moreover, we previously demonstrated that taurine incorporated into the progenitors of glutamatergic neurons, mainly pyramidal neurons, in the developing neocortex of fetal rats activates the with-no-lysine (WNK) protein kinase system that phosphorylates the neuron-specific type 2 K^+^-Cl^−^ cotransporter KCC2 (Inoue et al., 2012). The phosphorylation suppresses the Cl^−^ transporter activity of KCC2, thereby maintaining a high intracellular Cl^−^ level at ~30 mM made by the type 1 Na^+^-K^+^-2Cl^−^ cotransporter NKCC1 and confirming the depolarizing effect of the opening of GABA_A_ and glycine receptor Cl^−^ channels in these progenitors (Akita and Fukuda, 2020).

It has long been known that activation of GABA_A_ receptors on pyramidal neuron progenitors promotes differentiation of the progenitors and terminates their radial migration during fetal neocortical development (Wang and Kriegstein, 2009; Luhmann et al., 2015; Akita and Fukuda, 2020). We recently revealed that taurine plays important roles as an activator of GABA_A_ receptors in regulating both the differentiation (Tochitani et al., 2021) and the radial migration (Furukawa et al., 2014). About the differentiation, we found that the GABA_A_ receptor-mediated differentiation of progenitors from radial glia during early embryonic development was delayed by administration of an inhibitor of taurine synthesis, d-cysteine acid (d-CSA), to maternal mice or by knocking out the TauT gene (Tochitani et al., 2021). Similarly, about the radial migration, the acceleration of migration caused by blockade of GABA_A_ receptors was mimicked by the taurine depletion with d-CSA, in parallel with reduction of tonic GABA_A_ receptor currents in migrating progenitors (Furukawa et al., 2014). These findings indicated that the GABA_A_ receptor activation by taurine does facilitate progenitor differentiation and slow the speed of radial migration during normal fetal development. Nevertheless, the final proportion and destination of differentiated progenitors after migration were unaffected by taurine depletion, suggesting that a small amount of GABA in the fetal neocortex still has a significant role in these processes (Furukawa et al., 2014; Tochitani et al., 2021). The significance of taurine-mediated regulation of differentiation and migration in the following maturation of pyramidal neurons is yet unknown.

To examine the effect of taurine depletion during fetal and postnatal neocortical development on the functions of differentiated pyramidal neurons, here we used TauT knockout (KO) mice and compared intrinsic passive membrane properties and firing properties of layer II/III pyramidal neurons in the somatosensory cortex between wildtype (WT) and KO littermates at 3 weeks of postnatal age, using the whole-cell patch-clamp technique. We found that taurine depletion during development significantly altered the firing responses of pyramidal neurons to external stimuli.

## Materials and methods

### Ethical approval

All experiments were conducted according to the guidelines for Proper Conduct of Animal Experiments issued by Science Council of Japan, and the experimental procedures were reviewed and approved by the Institutional Animal Care and Use Committee of Hamamatsu University School of Medicine.

### Animals

Heterozygous (*Slc6a6*^+/−^; HT) and homozygous (*Slc6a6*^−/−^; Homo) TauT KO mice with a C57BL/6 background were produced as described previously (Ito et al., 2008; Tochitani et al., 2021). Six mice of each of the genotypes (WT, HT, and Homo) were used for electrophysiological experiments. These mice were littermates born to the same pairs of HT parents. This number of mice was minimal and sufficient to reach conclusions.

### Acute brain slice preparation

Coronal brain slices (300 μM in thickness) including the somatosensory cortex were obtained from male and female mice at the postnatal age of 19–23 days (P19-23). Mice were deeply anesthetized by intraperitoneal injection of pentobarbital and transcardially perfused with the cold (4°C) modified artificial cerebrospinal fluid (ACSF), whose composition was (in mM): 220 sucrose, 2.5 KCl, 1.25 NaH_2_PO_4_, 10 MgSO_4_, 0.5 CaCl_2_, 26 NaHCO_3_, 30 glucose, oxygenated with 95% O_2_/5% CO_2_. After full replacement of the blood with the ACSF, mice were decaptitated and brains were removed. Slices were made in the cold modified ACSF using a VT-1000 vibratome (Leica, Wetzlar, Germany) and incubated at room temperature for 1 h prior to experiments in the standard ACSF consisting of (in mM): 126 NaCl, 2.5 KCl, 1.25 NaH_2_PO_4_, 2 MgSO_4_, 2 CaCl_2_, 26 NaHCO_3_, 20 glucose bubbled with 95% O_2_/5% CO_2_.

### Electrophysiology

Electrophysiological recording of action potentials (APs) was done in layer II/III pyramidal neurons in the somatosensory cortex under current-clamp conditions with the whole-cell patch-clamp technique, using a MultiClamp 700B amplifier (Molecular Devices, San Jose, CA, United States). Membrane voltage records were lowpass filtered at 6 kHz and digitized at 20 kHz through a Digidata 1550 (Molecular Devices, San Jose, CA, United States). Recordings were made at 30°C in the standard ACSF supplemented with 10 μM CNQX, 50 μM d-AP5 and 50 μM picrotoxin to block AMPA- and NMDA-type glutamate receptors and GABA_A_ receptors, respectively. The drugs were purchased from Sigma-Aldrich. Patch electrodes were fabricated from borosilicate glass capillaries (1.5 mm in outer diameter; GD-1.5; Narishige, Tokyo, Japan) using a P-97 puller (Sutter Instrument, Novato, CA, United States). Electrodes were filled with the solution containing (in mM): 140 K-methanesulfonate, 10 KCl, 2 MgCl_2_, 10 HEPES, 3 Na_2_ATP, 0.2 NaGTP, 1 EGTA, pH 7.3, and had a pipette resistance of 3–4 MΩ. The liquid junction potential between the solutions was 4.8 mV and corrected online. The whole-cell patch was made onto the somata of pyramidal neurons under the guidance of infrared differential interference contrast imaging.

Resting membrane potential (RMP) was determined as the mean membrane voltage level for 300 ms before current injection. Cellular membrane capacitance around the soma (C_m_) was calculated from the time constant (τ) and the maximum amplitude of a membrane voltage change (ΔV) from RMP induced by injection of a hyperpolarizing current of −60 pA (I), using the equation τ = C_m_(ΔV/I), i.e., C_m_ = τI/ΔV. Input resistance was determined as the linear regression slope of the I-ΔV relationship obtained by injection of currents from −60 to +60 pA in 20 pA increments. Threshold voltage level for single AP generation was determined as the level from which the slope of voltage changes (dV/dt) became >3 times larger than the slope during injection of a 2 ms current pulse. The amplitude of a single AP spike evoked by the 2 ms current pulse was defined as the difference between the peak of the AP and either RMP or the most negative voltage level during afterhyperpolarization (AHP) if it existed. The amplitudes of repetitive APs during current injection for 1 s were defined as the differences between the peaks and the following minimum voltage levels during interspike intervals.

### Western blotting

The posterior halves of the cerebral cortex including the somatosensory area were collected from six WT and six Homo KO mice each. The cortices were homogenized and lysed in the lysis buffer containing (in mM) 150 NaCl, 50 Tris–HCl, 0.1% SDS, 1% NP-40, 0.5% sodium deoxycholate, added with protease inhibitors (#1697498; Roche, Basel, Switzerland). The supernatant after centrifugation was collected, separated by SDS-PAGE and transferred to a nitrocellulose membrane. Blots were blocked with 1% bovine serum albumin and incubated overnight at 4°C with the following primary antibodies: mouse anti-KCNB1 (Kv2.1) (1:1000, #SAB5200077, Sigma-Aldrich) or rabbit anti-KCNN2 (SK2) (1:1000, #APC-028, Alomone Labs, Jerusalem, Israel), and mouse anti-β actin (1:5000, #A5441, Sigma-Aldrich). The blots were then incubated with horseradish peroxidase-conjugated secondary antibody (GE Healthcare, Chicago, IL, United States) for 1 h at room temperature. Immunoblots were visualized with ECL (GE Healthcare) and photographed using a ChemiDoc Touch imaging system (Bio-Rad, Hercules, CA, United States). Band intensities of the blots were measured using Image Lab software (Bio-Rad).

### Statistics

Statistical comparisons were made using SPSS software (IBM, Armonk, NY, United States). Data normality was first assessed with the Kolmogorov–Smirnov test. When the normality was confirmed, multiple comparisons were made with one-way ANOVA followed *post hoc* by Ryan–Einot–Gabriel–Welsch F (REGW-F) or Dunnett’s T3 test, depending on whether equal variances could be assumed or not, respectively, from Levene statistic. When the normality was rejected, comparisons were made with the Kruskal–Wallis test (K-W) followed by the stepwise stepdown comparisons. The comparison of the proportions of neurons generating AHP after a single AP between genotypes was made with Pearson’s chi-square test. The amplitude and the peak time of AHP were compared only between HT and Homo neurons, because AHP was generated only in 3 of 24 WT neurons. The comparisons were made with Student’s *t* test or the Mann–Whitney *U* test, according to the data normality. Expression levels of Kv2.1 and SK2 measured by Western blotting were compared using the Mann–Whitney *U* test. *p* < 0.05 was considered significant. Data are presented as mean ± standard error of the mean (SEM).

## Results

### Broadening of single action potential spike in neocortical pyramidal neurons of TauT knockout mice

We previously confirmed that the taurine content in the telencephalon of TauT KO fetuses was reduced to 70% in HT and < 5% in Homo, compared with that in WT littermates, at embryonic day 12 (Tochitani et al., 2021). Since the capacity of taurine biosynthesis is still low in young animals (Hayes and Sturman, 1981), similar taurine levels would have been maintained in the brains of breast-fed infant mice (P19-23) used in this study. Indeed, in another TauT KO mouse line, the taurine content in the neocortex at 6–11 weeks of postnatal age was reported to be 57% in HT and 2% in Homo (Sergeeva et al., 2003). In the adult brain at 3 months age in our mouse line, the taurine content in Homo is increased to 15% (Ito et al., 2008).

**Figure 1:**
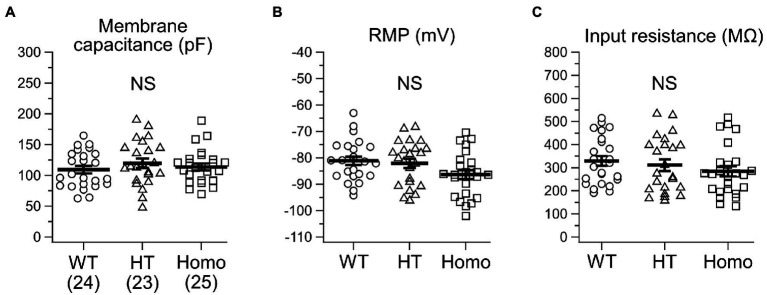
Passive membrane properties of somatosensory layer II/III pyramidal neurons in heterozygous (HT) and homozygous (Homo) TauT KO mice and wildtype (WT) littermates. **(A)** Comparison of cellular membrane capacitance around the soma. The numbers in brackets indicate the numbers of neurons analyzed. These are the same in all the figures except Figure 6 in this study. WT: 109.4 ± 6.1 pF, HT: 119.8 ± 7.6 pF, Homo: 113.9 ± 5.6 pF, *p* = 0.535 by ANOVA. **(B)** RMP. WT: −81.1 ± 1.6 mV, HT: −82.1 ± 1.8 mV, Homo: −86.3 ± 1.7 mV, *p* = 0.067 by ANOVA. **(C)** Input resistance. WT: 329.3 ± 21.2 MΩ, HT: 311.2 ± 25.1 MΩ, Homo: 284.8 ± 22.1 MΩ, *p* = 0.379 by ANOVA. NS, not significant. **(A–C)** Symbols indicate the values in individual neurons, and thick horizontal bars with error bars indicate means ± SEMs.

**Figure 2:**
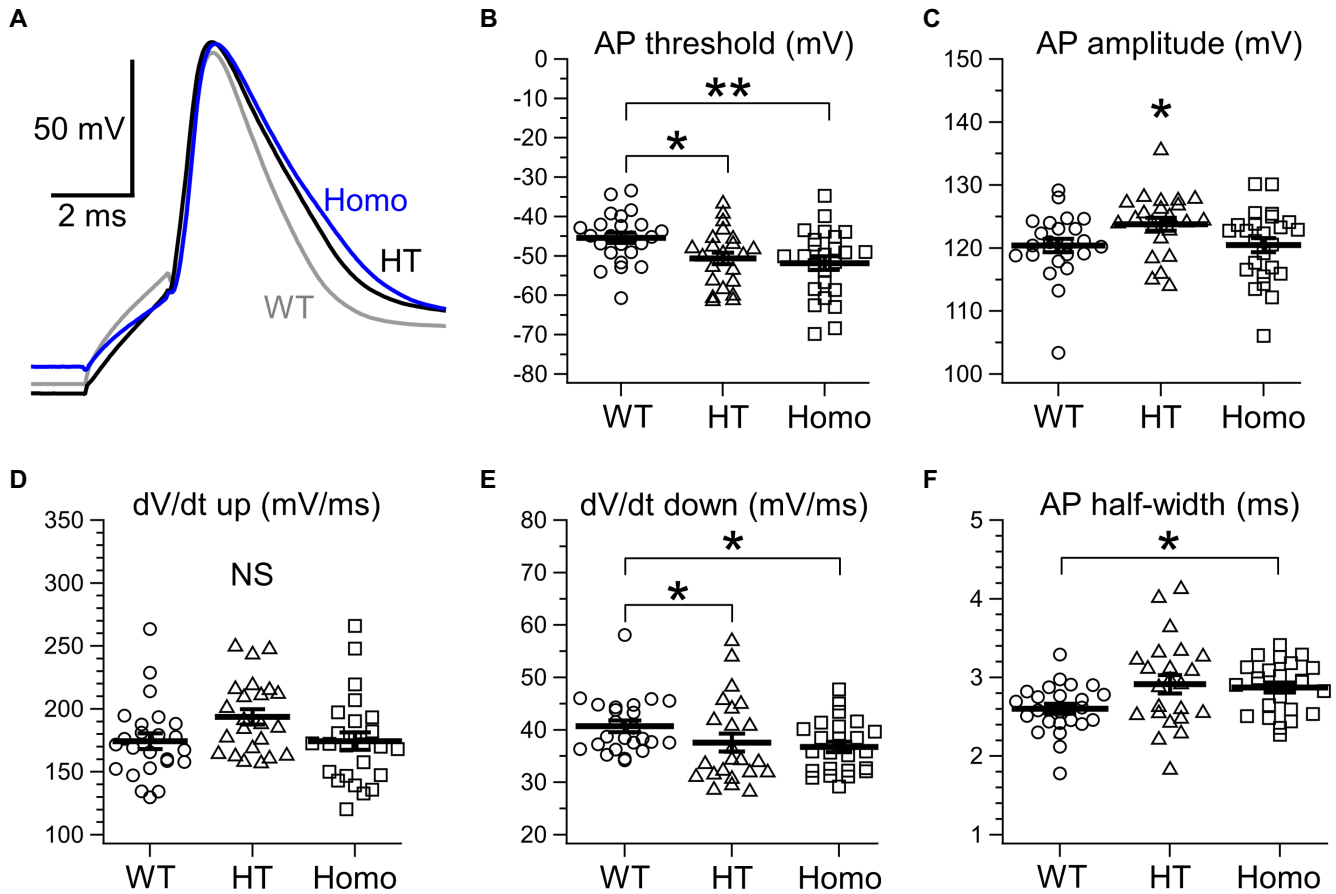
Properties of a single AP spike evoked by brief current injection. **(A)** Representative traces of a single AP evoked by a 2 ms current pulse. The gray trace obtained from a WT neuron, the black from a HT neuron and the blue from a Homo neuron are superimposed. **(B)** Threshold voltage level for an AP. *p < 0.05, **p < 0.01 by REGW-F. See text for the values of mean ± SEM. **(C)** AP spike amplitude. *p < 0.05 by REGW-F. See text for the values of mean ± SEM. **(D)** The maximum dV/dt during spike upstroke (dV/dt up). WT: 174.4 ± 6.2 mV/ms, HT: 193.7 ± 6.1 mV/ms, Homo: 174.4 ± 6.9 mV/ms; *p* = 0.062 by ANOVA. **(E)** The maximum negative dV/dt during spike downstroke (dV/dt down). Plots indicate absolute values of the dV/dt. *p < 0.05 by K-W. See text for the values of mean ± SEM. **(F)** The width at half-maximal spike amplitude. *p < 0.05 by Dunnett’s T3. See text for the values of mean ± SEM. **(B–F)** Symbols indicate the values in individual neurons, and thick bars with error bars indicate means ± SEMs.

Membrane voltage changes induced by current injection under current-clamp conditions were analyzed in neocortical somatosensory layer II/III pyramidal neurons in acute brain slice preparations. About passive membrane properties of neurons, we found no significant differences in cellular membrane capacitance around the soma (Figure 1A), RMP (Figure 1B) and input resistance at around RMP (Figure 1C) between genotypes, although some decreasing trends in RMP and input resistance were seen in KO neurons. About a single AP spike evoked by a 2 ms brief current pulse, we found broadening of the spike in KO neurons (Figure 2A). The half-width of the spike in Homo neurons was significantly prolonged, compared with WT neurons (WT: 2.60 ± 0.06 ms, *n* = 24 from 6 mice, HT: 2.91 ± 0.12 ms, *n* = 23 from 6 mice, Homo: 2.87 ± 0.06 ms, *n* = 25 from 6 mice; *p* = 0.013, WT vs. Homo; *p* = 0.074, WT vs. HT by Dunnett’s T3; Figure 2F). This was due to the reduction in speed of spike downstroke (dV/dt down; WT: −40.7 ± 1.1 mV/ms, HT: −37.6 ± 1.7 mV/ms, Homo: −36.8 ± 1.0 mV/ms; *p* = 0.033, WT vs. HT and Homo by K-W; Figure 2E) without significant changes in speed of spike upstroke (dV/dt up; Figure 2D). In addition to the broadening, the threshold voltage level for spike generation was significantly lowered in KO neurons (WT: −45.4 ± 1.3 mV, HT: −50.6 ± 1.5 mV, Homo: −51.8 ± 1.7 mV; *p* = 0.035, WT vs. HT and Homo by REGW-F; Figure 2B). The AP amplitude was slightly larger only in HT neurons (WT: 120.4 ± 1.0 mV, HT: 123.8 ± 1.0 mV, Homo: 120.5 ± 1.1 mV; *p* = 0.048, HT vs. WT and Homo by REGW-F; Figure 2C). The AHP of a single AP (not shown) was generated only in 3 of 24 WT neurons, whereas it was noticeable in 8 of 23 HT neurons and in 8 of 25 Homo neurons. However, the chi-square test did not indicate significant differences in the proportion of AHP-positive neurons between genotypes (*p* = 0.163). The amplitude of AHP (WT: −0.60 ± 0.18 mV, HT: −0.44 ± 0.09 mV, Homo: −0.92 ± 0.25 mV; *p* = 0.100, HT vs. Homo by *t* test) and the peak time of AHP from the AP spike peak (WT: 340.0 ± 115.9 ms, HT: 236.2 ± 35.0 ms, Homo: 488.5 ± 193.9 ms; *p* = 0.574, HT vs. Homo by Mann–Whitney *U* test) were similar between genotypes.

### Lower frequency of repetitive action potential firing during moderate current injection in TauT knockout pyramidal neurons

**Figure 3:**
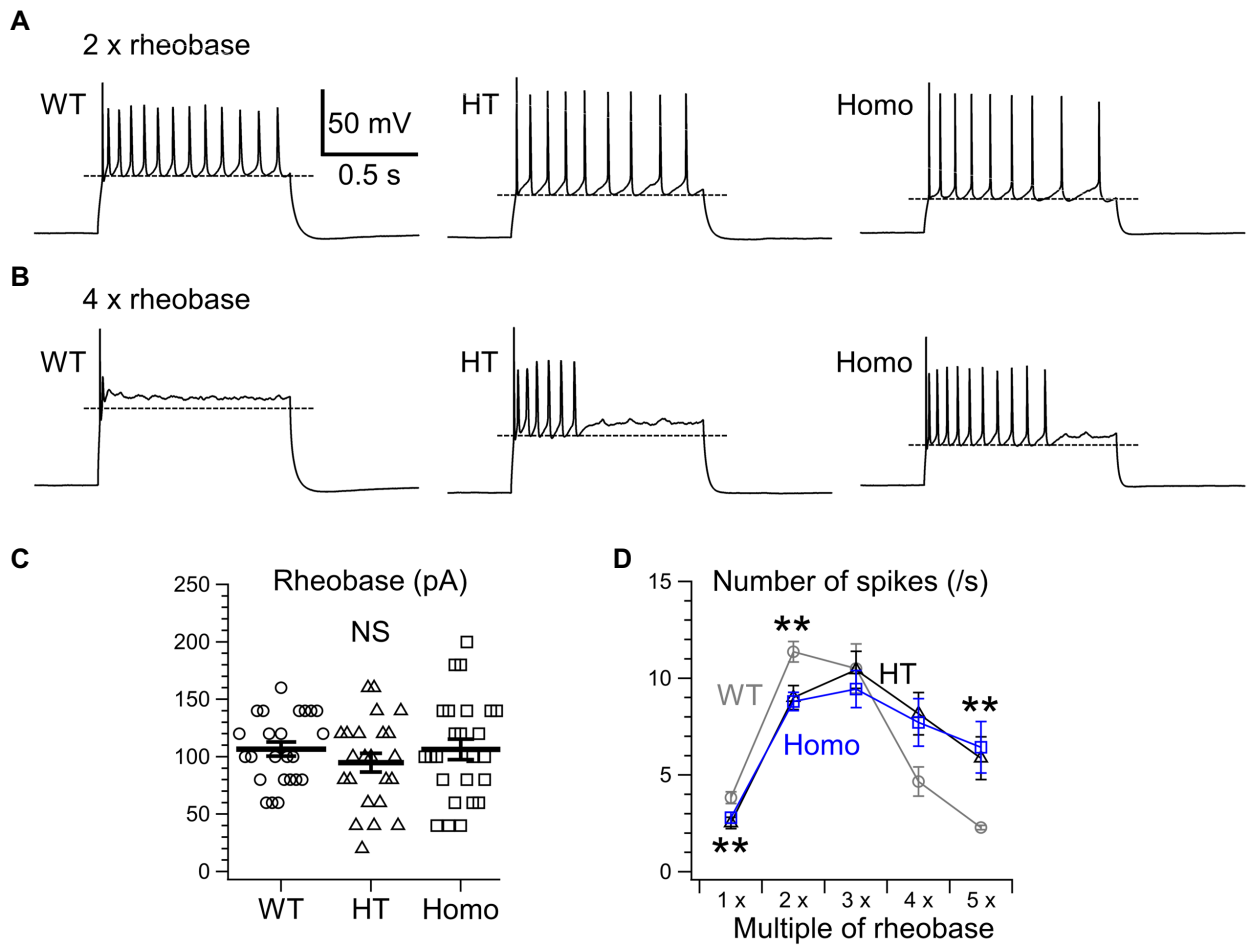
Repetitive AP firing induced by current injection for 1 s. **(A)** Representative traces of repetitive firing induced by injection of two times the rheobase current (2 × rheobase) in WT, HT, and Homo neurons. **(B)** The traces induced by 4 × rheobase in the same neurons as in **(A)**. Broken lines in **(A,B)** indicate mean minimum membrane voltage levels during interspike intervals in these examples. **(C)** Rheobase was determined by applying current steps in 20 pA increments. WT: 106.7 ± 6.1 pA, HT: 94.8 ± 8.2 pA, Homo: 106.4 ± 8.9 pA, *p* = 0.485 by ANOVA. Symbols indicate the values in individual neurons, and thick bars with error bars indicate means ± SEMs. NS, not significant. **(D)** The numbers of AP spikes induced by 1–5 × rheobase for 1 s in WT (gray), HT (black), and Homo (blue) neurons. Symbols with error bars indicate means ± SEMs. **p < 0.01, WT vs. HT and Homo by K-W.

**Figure 4:**
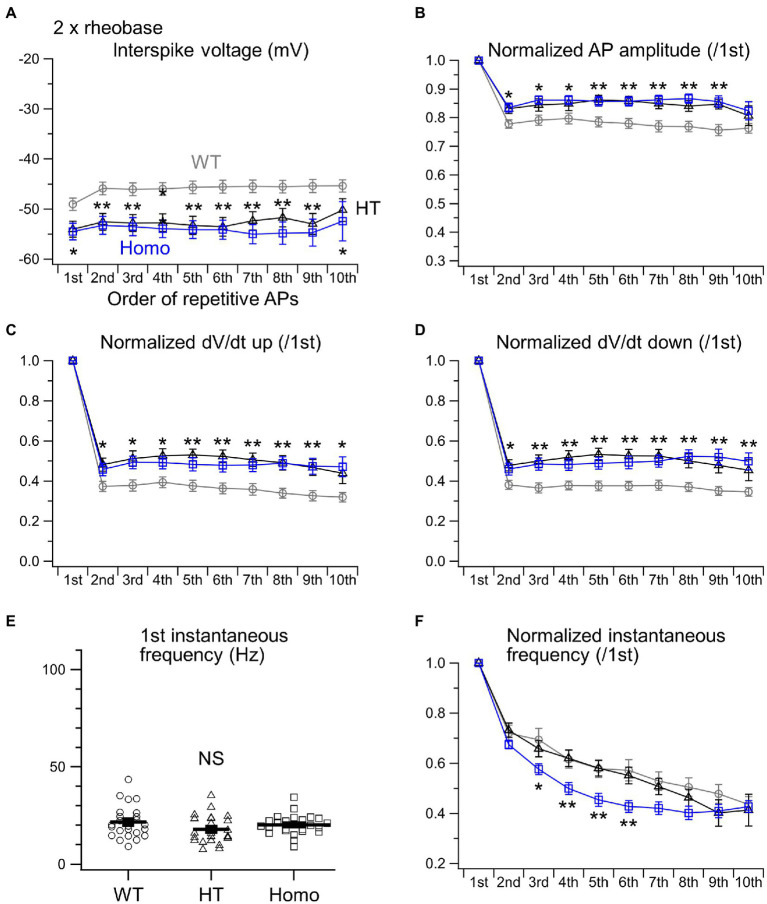
Properties of repetitive AP firing induced by 2 × rheobase. **(A)** Interspike voltage levels were defined as the minimum membrane voltage levels between adjacent AP spikes. The initial 10 interspike voltages were compared between WT (gray), HT (black) and Homo (blue) neurons. **(B)** The amplitudes of initial 10 APs were normalized to that of the first AP. **(C)** dV/dt ups of initial 10 APs were normalized to the first. **(D)** dV/dt downs of initial 10 APs were normalized to the first. *p < 0.05, **p < 0.01, WT vs. HT and Homo by REGW-F. **(E)** Comparison of the first instantaneous frequency. Symbols indicate the values in individual neurons, and thick bars with error bars indicate means ± SEMs. WT: 21.7 ± 1.7 Hz, HT: 17.9 ± 1.5 Hz, Homo: 20.2 ± 1.0 Hz, *p* = 0.184 by ANOVA. NS, not significant. **(F)** The initial 10 instantaneous frequencies were normalized to the first. * at 3rd, *p* < 0.05, WT vs. HT and Homo by K-W. ** at 4th, *p* < 0.01, WT vs. HT and Homo by Dunnett’s T3. ** at 5th, *p* < 0.01, WT vs. HT and Homo by REGW-F. ** at 6th, *p* < 0.01, WT vs. HT and Homo by K-W.

We next examined the properties of repetitive AP firing induced by current injection for 1 s. The lower AP threshold level in KO neurons (Figure 2B) had implications for enhanced excitability in these neurons. Nevertheless, the minimum strength of the current required for eliciting at least one AP, called rheobase, did not differ between genotypes (Figure 3C). This means that the membrane excitability under resting conditions was not significantly altered by the lower threshold level in KO neurons. When repetitive AP firing was induced by injection of two times the rheobase current (2 × rheobase), the number of spikes in HT and Homo neurons was significantly smaller than that in WT neurons, despite no difference between HT and Homo neurons (WT: 11.4 ± 0.5 spikes/s, HT: 9.0 ± 0.6 spikes/s, Homo: 8.8 ± 0.5 spikes/s; *p* = 0.001, WT vs. HT and Homo by K-W; Figures 3A,D). This reduction in firing frequency in HT and Homo neurons was accompanied by lower membrane voltage levels during interspike intervals in these neurons, compared with WT neurons (e.g., the 5th interspike minimum voltage level, WT: −45.6 ± 1.3 mV, HT: −53.2 ± 1.8 mV, Homo: −54.1 ± 1.8 mV; *p* = 0.001, WT vs. HT and Homo by REGW-F; Figures 3A, 4A). The spike amplitude, dV/dt up and dV/dt down of APs were reduced during repetitive firing in all genotypes, and we found that all the reductions in HT and Homo neurons were significantly smaller than those in WT neurons (Figures 4B–D). The smaller reductions resulted in smaller increases in spike half-width during repetitive firing despite the wider first AP in KO neurons than in WT neurons (Supplementary Figure S1A). These indicate that the lower interspike membrane voltage levels in KO neurons allowed voltage-gated Na^+^ channels (Nav) to recover more from inactivation that occurred at AP peaks, resulting in larger repetitive APs, which in turn activated more the voltage-gated K^+^ channels (Kv) that caused spike repolarization, resulting in narrower repetitive APs. Spike frequency adaptation was assessed as the gradual reduction in instantaneous frequency (the reciprocal of the interspike interval) during repetitive firing. The first instantaneous frequency, i.e., the reciprocal of the interval between the first and the second APs, did not significantly differ between genotypes (Figure 4E), although the mean value of the frequency in HT neurons was somewhat smaller than the others. The frequency reduction during repetitive firing, i.e., the adaptation, was found to be significantly faster in Homo neurons than the others (Figure 4F). Thus, these results imply that the K^+^ conductance during interspike intervals would have been more increased in TauT KO neurons, thereby counteracting more strongly the depolarization caused by current injection and delaying repetitive AP generation. For the AHP generated after the end of current injection, there were no significant differences in its amplitude between genotypes (WT: −2.65 ± 0.23 mV, HT: −2.03 ± 0.23 mV, Homo: −2.44 ± 0.42 mV; *p* = 0.362 by ANOVA).

### Sustained repetitive action potential firing during strong current injection in TauT knockout neurons

**Figure 5:**
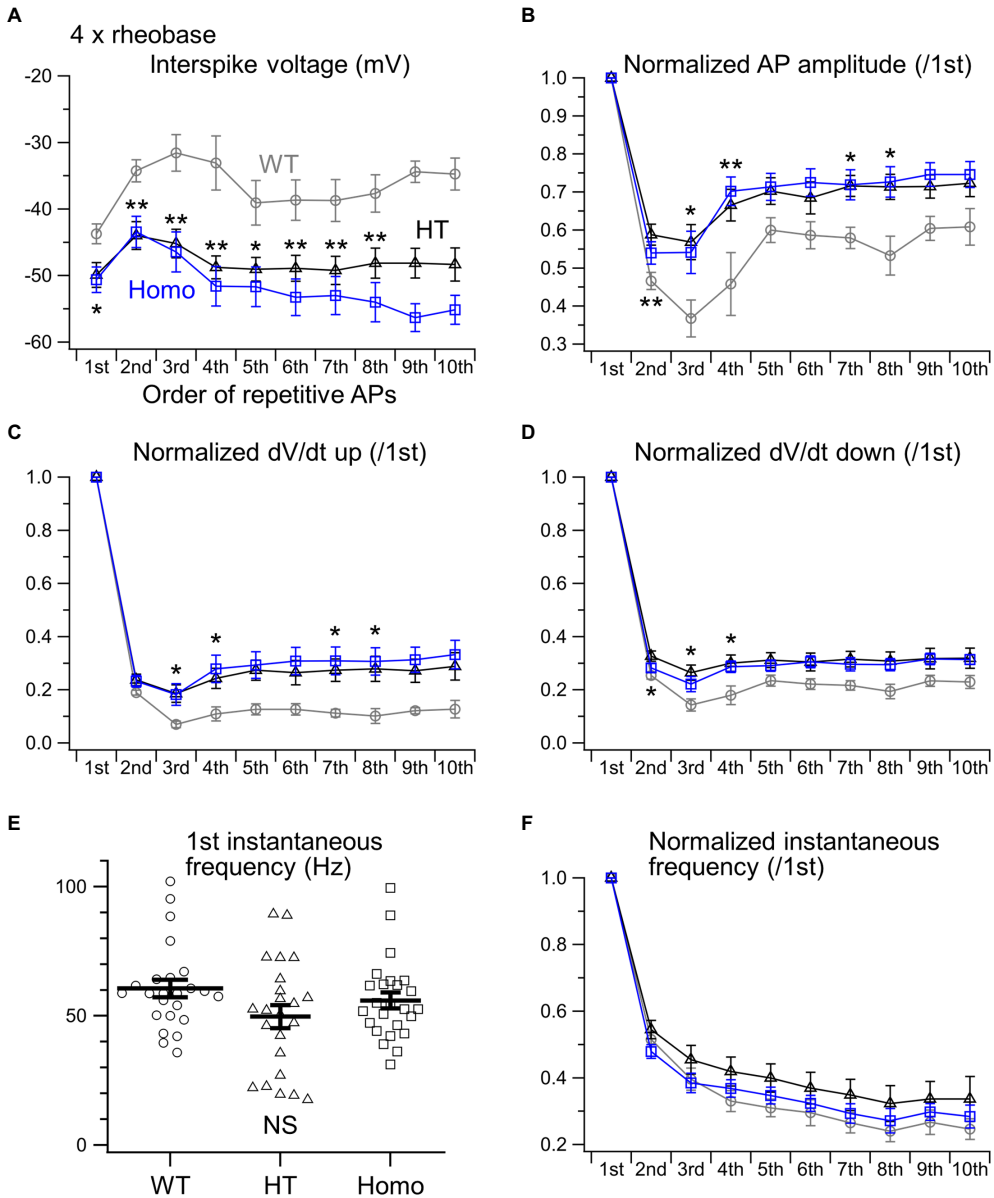
Properties of repetitive AP firing induced by 4 × rheobase. **(A)** The initial 10 interspike voltage levels were compared between WT (gray), HT (black) and Homo (blue) neurons. **(B)** Normalized AP amplitudes of the initial 10 APs. **(C)** Normalized dV/dt ups. **(D)** Normalized dV/dt downs. *p < 0.05, **p < 0.01, WT vs. HT and Homo by REGW-F. **(E)** The first instantaneous frequency. Symbols indicate the values in individual neurons, and thick bars with error bars indicate means ± SEMs. WT: 60.6 ± 3.4 Hz, HT: 49.7 ± 4.5 Hz, Homo: 55.9 ± 3.1 Hz, *p* = 0.220 by K-W. NS, not significant. **(F)** The initial 10 instantaneous frequencies were normalized to the first. **(A–D,F)** The parameters of the 9th and 10th APs were not compared statistically, because the number of spikes induced by 4 × rheobase in WT neurons was so small (4.7 ± 0.8 spikes/s) that the sample sizes of the 9th and 10th APs in WT neurons were less than 5.

When much stronger currents of 4 × and 5 × rheobase were injected into WT neurons, the injection produced less than five of rapidly damping APs at the beginning of the injection, whereas the same current injection into HT and Homo neurons produced much larger numbers of repetitive APs (WT: 2.3 ± 0.1 spikes/s, HT: 5.9 ± 1.1 spikes/s, Homo: 6.4 ± 1.3 spikes/s induced by 5 × rheobase; *p* = 0.004, WT vs. HT and Homo by K-W; Figures 3B,D) for longer periods. These differences in spike number were accompanied by much higher membrane voltage levels during interspike intervals in WT neurons, compared with KO neurons (Figures 3B, 5A). The reductions in spike amplitude, dV/dt up and dV/dt down and the increase in spike half-width during repetitive firing were still clearly smaller in KO neurons (Figures 5B–D; Supplementary Figure S1B). The first instantaneous frequency and the spike frequency adaptation under this condition were similar between genotypes (Figures 5E,F). The AHP amplitude after the end of current injection were also similar (WT: −2.64 ± 0.27 mV, HT: −1.91 ± 0.23 mV, Homo: −2.42 ± 0.45 mV; *p* = 0.320 by ANOVA). These results are consistent with the idea that the more increased K^+^ conductance during interspike intervals in TauT KO neurons produced lower membrane voltage levels during the intervals and promoted greater recovery of Nav from inactivation, and that this resulted in more sustained repetitive AP firing in KO neurons than in WT neurons, in which the firing was rapidly abolished through the inactivation, during strongly depolarizing current injection.

### Similar protein expression levels of Kv2.1 and SK2 channels in the neocortex of wildtype and TauT knockout mice

**Figure 6:**
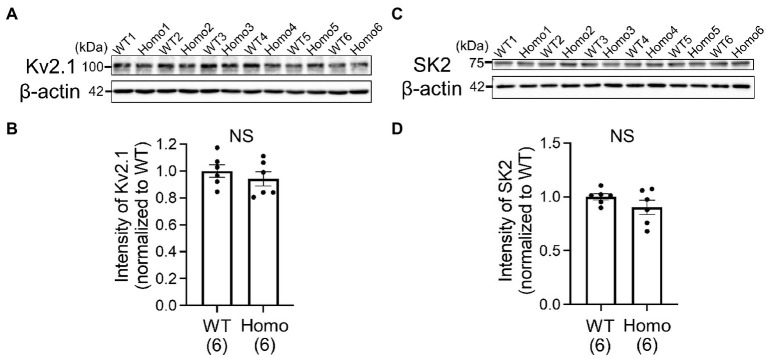
Comparison of protein expression levels of Kv2.1 and SK2 channels in the neocortex of WT and TauT Homo KO mice by Western blot analysis. **(A)** Immunoblots indicating the levels of Kv2.1 and β-actin in individual neocortices of WT and Homo KO mice. Lysates from six WT (WT1-WT6) and six Homo (Homo1-Homo6) mice were loaded onto the same polyacrylamide gel and blotted onto the same membrane. **(B)** Statistical comparison of Kv2.1 levels between WT and Homo mice. The ratio of Kv2.1 band intensity to β-actin band intensity in each lane was normalized to the mean of the ratios in the six WT lanes. Normalized ratios are plotted for each group of WT and Homo, with the height of the bar representing the mean in each group. Error bars indicate SEMs. The numbers in brackets indicate the numbers of mice analyzed. *p* = 0.394 by Mann–Whitney *U* test. NS, not significant. **(C)** Immunoblots for SK2 made in the same way as in **(A)**. **(D)** Comparison of SK2 levels. The ratio of SK2 intensity to β-actin intensity in each lane was normalized to the mean of the ratios in the six WT lanes. *p* = 0.394 by Mann–Whitney *U* test.

Candidate K^+^ channels responsible for the K^+^ conductance during interspike intervals in neocortical pyramidal neurons are delayed rectifier Kv2 channels (Guan et al., 2013) and Ca^2+^-activated SK-type K^+^ channels (Gill and Hansel, 2020). Among these, we focused on the Kv2.1 and SK2 subtypes, which have been reported to be expressed in layer II/III (Gymnopoulos et al., 2014; Bishop et al., 2015), and compared their protein expression levels in WT and TauT KO mice. However, Western blotting of lysates from the posterior half of the neocortex showed no clear differences in the expression levels of both Kv2.1 and SK2 between WT and Homo KO mice (Figure 6).

## Discussion

In this study, we found that taurine depletion during fetal and postnatal development caused by knockout of TauT significantly altered the intrinsic firing properties of neocortical layer II/III pyramidal neurons in the somatosensory cortex. The main finding was that, in TauT KO neurons, the frequency of repetitive AP firing induced by moderate stimulation with 2 × rheobase was lower than that in WT neurons, whereas the firing was sustained even during strongly depolarizing stimulation with >4 × rheobase, by which the firing in WT neurons was rapidly abolished due to Nav inactivation. These differences between KO and WT neurons were made by the lower membrane voltage levels during interspike intervals of repetitive APs in KO neurons. The lower interspike voltage levels delay the subsequent Nav activation and also promote greater recovery of Nav from inactivation occurring at AP peaks. The larger the proportion of recovered Nav, the faster spike upstrokes and the larger amplitudes of subsequent APs are generated, and these further enhance the opening of Kv responsible for spike downstroke. These were indeed indicated by the smaller reductions of these parameters during repetitive firing in KO neurons than in WT neurons. Therefore, the taurine depletion was found to blunt the responses of pyramidal neurons to external stimuli through increasing the stability of repetitive firing, presumably mediated by larger increases in membrane K^+^ conductance during interspike intervals.

Despite the significantly lower threshold voltage level for single AP generation in KO neurons, the rheobase in these neurons was not smaller than that in WT neurons. This implies that the threshold reduction was so small and/or offset by different factors. Rheobase is determined not only by the AP threshold, but also by RMP and input resistance. Although the differences in RMP and input resistance between genotypes did not reach statistical significance, there were the trends of lower RMP and lower input resistance in KO neurons compared with WT neurons (Figures 1B,C). Because both the trends are the factors increasing rheobase, the trends might have canceled out the effect of the lower AP threshold in KO neurons.

The membrane voltage levels during interspike intervals in cortical pyramidal neurons are mainly determined by the activities of delayed rectifier Kv2 channels (Guan et al., 2013; Bishop et al., 2015) and Ca^2+^-activated SK- and KCa3.1-types of K^+^ channels (Gymnopoulos et al., 2014; Gill and Hansel, 2020; Roshchin et al., 2020), all of which do not open under resting conditions and open after AP peaks. Therefore, these K^+^ channel activities would be enhanced without affecting the input resistance under resting conditions in TauT KO neurons. The comparable protein expression levels of Kv2.1 and SK2, representative subtypes of the K^+^ channels, in WT and TauT KO neocortex suggest the possibility that expression levels of other subtypes such as Kv2.2 (Bishop et al., 2015), SK1, SK3 (Gymnopoulos et al., 2014) and KCa3.1 (SK4 or IK; Roshchin et al., 2020) might be elevated in TauT KO neurons. It is also possible that the voltage dependence, Ca^2+^ dependence or both of these K^+^ channels in TauT KO neurons may differ from WT neurons due to differences in the phosphorylation state of the channels (Bishop et al., 2015; Gill and Hansel, 2020). On the other hand, the lower AP threshold and the spike broadening of isolated single APs in KO neurons suggest the reduced activities of other types of K^+^ channels, like Ca^2+^-activated BK (Bock and Stuart, 2016), Kv1 (Kole et al., 2007) and Kv4 (Carrasquillo and Nerbonne, 2014). Therefore, it is very likely that multiple types of K^+^ channels are differentially modulated in TauT KO neurons. The mechanisms by which taurine depletion induces such modulation need further investigation. One possibility is the reduced signaling through the WNK kinase system under taurine-depleted conditions, because the WNK kinase and its downstream kinases of the SPS1-related proline/alanine-rich kinase (SPAK) and the oxidative stress-responsive kinase 1 (OSR1) have been reported to regulate several types of K^+^ channels in other tissues (Elvira et al., 2016; Taylor et al., 2018; Bi et al., 2020). In our recent microarray assay of signal transduction proteins in WT and TauT Homo KO brains (Watanabe et al., 2022), the protein expression levels of the major WNK isoforms WNK1, WNK2 and WNK3, and the phosphorylation level of WNK1 at serine 382 were comparable between them (Supplementary Table S1), although the phosphorylation levels of the other WNK isoforms were not investigated. Furthermore, the assay revealed significantly increased expression levels of 17 proteins and phosphorylation levels of 4 proteins in TauT KO brains (Supplementary Table S1; Watanabe et al., 2022). Of these, the increased phosphorylation level of STAT3 (signal transducer and activator of transcription 3) at tyrosine 705 was particularly noticeable. STAT3 is involved in leptin signaling in the central nervous system and regulates energy metabolism in animals (Liu et al., 2021). Further studies are needed to determine whether this and the other signaling proteins may modulate K^+^ channel activity in pyramidal neurons. The broadening of AP spikes with maintained peak AP levels increases the amount of Ca^2+^ entry and the subsequent Ca^2+^-induced Ca^2+^ release from sub-plasma membrane ryanodine receptors during the spikes (Akita and Kuba, 2000; Sahu et al., 2019). The resulting enhancement of intracellular Ca^2+^ rises would contribute to the increased activity of Ca^2+^-activated K^+^ channels during the initial AP generation in TauT KO neurons. During subsequent repetitive AP spikes, the broadening was greater in WT neurons, but this was accompanied by greater reductions in AP amplitude. The amplitude reduction reduces the Ca^2+^ entry, and this would have led to weaker activation of Ca^2+^-activated K^+^ channels during repetitive APs in WT neurons than in TauT KO neurons.

The intensity of sensation to sensory stimuli is encoded by firing frequency. Therefore, the stimulus range < 3 × rheobase in this study, in which the firing rate increased with increasing current, could be regarded as the physiological range of sensory stimuli. In this range, our results imply that taurine depletion during brain development reduces the sensitivity of mice to somatosensory stimuli. In other words, taurine action in the developing brain plays a role in enhancing the somatosensory sensitivity. If the firing frequency of pyramidal neurons in other neocortical areas is also reduced in TauT KO mice, it might be reflected as the dullness or slowness of other neocortical functions, such as thinking or motor execution, of the KO mice. Indeed, our recent behavioral analysis of the KO mice revealed that, during the elevated plus maze test, Homo KO mice spent significantly less time in the closed arm and more time in the center region, with a tendency of spending more time in the open arm, although the total distance of movement during the test was similar between genotypes (Watanabe et al., 2022). This indicated that KO mice exhibited reduced anxiety-like behavior and had difficulty making judgements regarding potential risk assessment, suggesting blunted frontal lobe function. Nevertheless, conventional TauT KO mice used in this and our recent studies show a variety of impairments in many organs including skeletal muscles (Warskulat et al., 2007; Ito et al., 2008, 2014, 2015; Qvartskhava et al., 2019; Watanabe et al., 2022). Therefore, it is difficult to precisely assess the impact of altered pyramidal neuron responses through behavioral analysis of the mice. For that purpose, conditional KO mice of TauT in pyramidal neurons would be useful.

The differences in firing properties between HT and Homo neurons were not significant in this study. This means that only 30–40% reduction in taurine content had nearly the maximum effect on the firing properties. Thus, some low-affinity taurine-sensing system would be involved in the firing regulation. Our results suggest the possibility that dietary restriction of taurine during the fetal and infantile period may cause such a reduction in taurine content that it significantly reduces the sensitivity of pyramidal neurons to external stimuli, especially in the species with a low capacity for taurine biosynthesis, like humans (Hayes and Sturman, 1981).

Studies on the effects of taurine depletion on neocortical neurons at the cellular level were largely limited to their developmental period (Furukawa et al., 2014; Kilb and Fukuda, 2017; Tochitani et al., 2021). Therefore, this study firstly reports one of the consequences of taurine depletion during development on the properties of differentiated neocortical neurons. The more stable repetitive firing of TauT KO neurons may suggest the possibility of its antiepileptic effect. This idea appears to be controversial, given the partial agonist action of taurine on GABA_A_ and glycine receptors. Effects of taurine on epileptic seizures have long been discussed, but there have been both types of reports supportive and unsupportive of its antiepileptic effects (Oja and Saransaari, 2013). In addition, we have never seen spontaneous seizures in our TauT KO mice. The properties of interneurons, synaptic connections and glial contributions are also involved in epileptogenesis, and thus the assessment of these properties in TauT KO mice is necessary. In the striatum of another TauT KO mouse line, reduced agonist sensitivity of synaptic and extrasynaptic GABA_A_ receptors was reported (Sergeeva et al., 2007), although it was not examined in the neocortex. We are now striving to identify other properties of neocortical neurons in our TauT KO mice, and we will report them elsewhere.

## Data availability statement

The original data on which the figures in this article are based can be made available by the corresponding authors upon reasonable request.

## Ethics statement

The animal study was reviewed and approved by the Institutional Animal Care and Use Committee of Hamamatsu University School of Medicine.

## Author contributions

YH, TA, HM, and AF conceived and designed the study. TI produced TauT knockout mice. YH performed electrophysiological experiments. YH and TA analyzed the data. MW performed Western blotting. TA wrote the manuscript. All authors contributed to the article and approved the submitted version.

## Funding

This work was supported by Grants-in-Aid for Scientific Research (B) (#25293052, #21H02661), and for Challenging Exploratory Research (#24659508) from the Japan Society for the Promotion of Science, and Grants-in-Aid for Scientific Research on Innovative Areas (Sugar Chain and Neuronal Functions #26110705 and Neuro-oscillology #15H05872) from the Ministry of Education, Culture, Sports, Science and Technology of Japan.

## Conflict of interest

The authors declare that the research was conducted in the absence of any commercial or financial relationships that could be construed as a potential conflict of interest.

## Publisher’s note

All claims expressed in this article are solely those of the authors and do not necessarily represent those of their affiliated organizations, or those of the publisher, the editors and the reviewers. Any product that may be evaluated in this article, or claim that may be made by its manufacturer, is not guaranteed or endorsed by the publisher.
