# Supplementary material for: Taurine depletion during fetal and postnatal development blunts firing responses of neocortical layer II/III pyramidal neurons
Source: Front Mol Neurosci. 2022 Nov 17;15:806798. doi: 10.3389/fnmol.2022.806798 (PMC9712787; doi:10.3389/fnmol.2022.806798)
Supplement: Supplementary file 1 [file Data_Sheet_1.PDF]

Supplementary Material

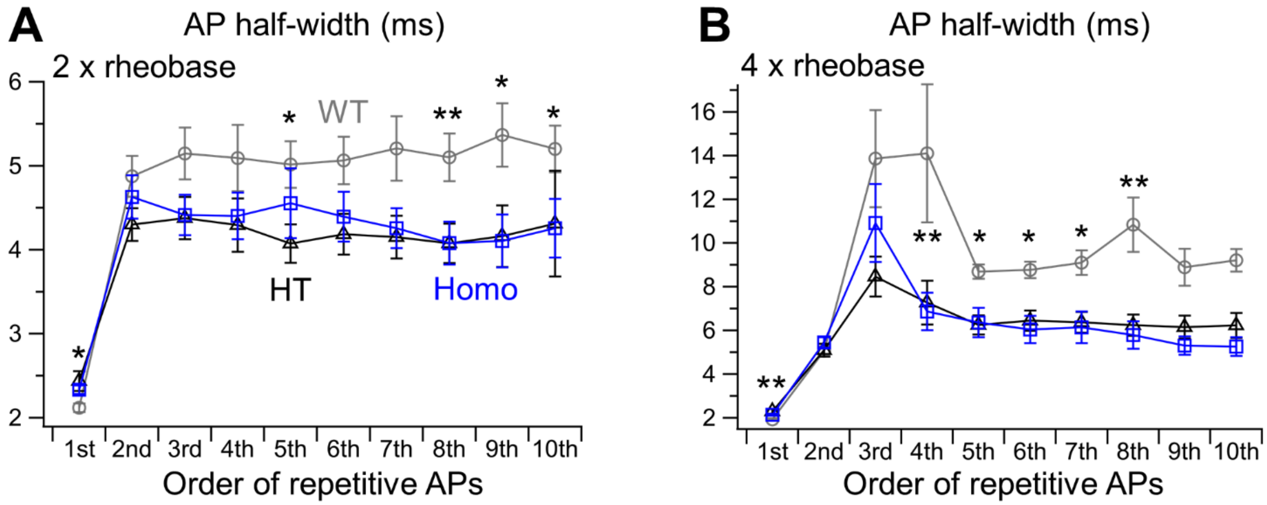

Supplementary Figure 1. Changes in half-width of repetitive APs during current injection for 1 s. (**A**) The half-widths of the first 10 APs evoked by 2 × rheobase were compared between WT (gray), HT (black) and Homo (blue) neurons. * at 1st, $p < 0.05$, WT vs Homo by Dunnett's T3. * at 5th, $p < 0.05$, WT vs HT by K-W. ** at 8th, $p < 0.01$, WT vs HT & Homo by REGW-F. * at 9th, $p < 0.05$, WT vs HT & Homo by REGW-F. * at 10th, $p < 0.05$, WT vs HT & Homo by K-W. (**B**) Comparison of the half-widths of the first 10 APs evoked by 4 × rheobase. ** at 1st, $p < 0.01$, WT vs HT & Homo by Dunnett's T3. ** at 4th, $p < 0.01$, WT vs HT & Homo by K-W. * at 5th-7th, $p < 0.05$, WT vs HT & Homo by K-W. ** at 8th, $p < 0.01$, WT vs HT & Homo by REGW-F. The half-widths of the 9th and 10th APs were not compared statistically due to small sample sizes.

Supplementary Methods

Protein microarray

Expression profiles of signal transduction proteins in the WT and TauT Homo KO neocortex were examined using the Kinex KAM-880 Antibody Microarray service (Kinexus Bioinformatics, Vancouver, BC, Canada). Some of the results have already been reported in our recent paper (Watanabe et al., 2022) and all the analysis results are listed in **Supplementary Table 1**. In the microarray, we compared the expression levels and phosphorylation status of signaling proteins targeted by 518 pan-specific antibodies (for total protein expression) and 359 phosphorylation site-specific antibodies (for phosphorylation) between the two genotypes. Details of the method were described in our previous report (Watanabe et al., 2022). In **Supplementary Table 1**, differences in expression and phosphorylation levels are shown as percent changes in those levels in TauT KO samples relative to control WT samples (%CFC; changes from control), defined as [(background-corrected signal intensity of TauT KO sample / background-corrected signal intensity of WT sample) × 100] – 100. Based on the absolute value of %CFC > 50, the %Error Range in duplicate measurements, and the intensity of the background-corrected (Globally Normalized) averaged value, the levels of target proteins labeled "Best" can be considered significantly different between Taut KO and WT neocortices.
